# The Degree of Stent Apposition Measured by Stent Enhancement at the Level of the Side Branch as a Novel Predictor of Procedural Success in Left Main PCI

**DOI:** 10.3390/jpm13050791

**Published:** 2023-05-03

**Authors:** Ștefan Dan Cezar Moț, Adela Mihaela Șerban, Alexandra Dădârlat-Pop, Raluca Tomoaia, Dana Pop

**Affiliations:** 1 Cardiology Department, Heart Institute Niculae Stăncioiu, 19-21 Motilor Street, 400001 Cluj-Napoca, Romania; 2 5th Department of Internal Medicine, Faculty of Medicine, “Iuliu Hatieganu” University of Medicine and Pharmacy, 400012 Cluj-Napoca, Romania; 3 Department of Cardiology, Clinical Rehabilitation Hospital, 400347 Cluj-Napoca, Romania

**Keywords:** left main, stent enhancement, stent apposition, side branch, kissing, bifurcation

## Abstract

Background: Stent enhancement techniques allow adequate visualization of stent deformation or incomplete stent expansion at the ostium of the side branch. Measuring the stent enhancement side branch length (SESBL) could reflect procedural success in terms of optimal stent expansion and apposition with better long-term outcomes. A longer SESBL may reflect a better stent apposition at the polygon of confluence and at the side branch (SB) ostium. Methods: We evaluated 162 patients receiving the left main (LM) provisional one-stent technique and measured the SESBL, dividing them into two groups: SESBL≤ 2.0 mm and SESBL > 2.0 mm. Results: The mean SESBL was 2.0 ± 1.2 mm. More than half of the bifurcations had both main and side branch lesions (Medina 1-1-1) (84 patients, 51.9%) and the length of the SB disease was 5.2 ±1.8 mm. Kissing balloon inflation (KBI) was performed in 49 patients (30.2%). During follow-up (12 months), there was a significantly higher rate of cardiac death in the SESBL ≤ 2.0 mm group (*p* = 0.02) but no significant difference in all major adverse cardiovascular events (MACEs) (*p* = 0.7). KBI did not influence the outcomes (*p* = 0.3). Conclusion: Suboptimal SESBL is positively correlated with worse outcomes and SB compromise. This novel sign could aid the LM operator to assess the level of stent expansion at the ostium of the SB in the absence of intracoronary imaging.

## 1. Introduction

Due to its major importance in terms of outcomes and the amount of myocardium involved, percutaneous coronary intervention (PCI) for left main (LM) has been continuously standardized and improved by expert joint committees [1,2,3,4]. The guidelines and consensuses strived for uniform distribution of the quality of the operative act among interventional cardiology centers by introducing some “minimum” but mandatory requirements: a minimum annual number of LM procedures per operator, knowledge of stent platform proprieties, knowledge of bifurcation techniques, adequate lesion preparation, use of intracoronary imaging, avoidance of longitudinal stent crush, avoidance of stent undersizing, underexpansion and malapposition, mandatory performance of proximal optimization technique (POT) and correct use of kissing balloon inflation (KBI) [3,4,5].

All these steps have resulted in significant progress for this particular procedure, with better long-term clinical results, reflected in numerous studies and meta-analyses [6,7,8]. Outcomes were improved, but there are still many debates on some aspects. For example, the POT step is considered to be mandatory while KBI is considered to be optional. This is mainly because of the current data showing significant mortality hazard when not using POT but no clear benefit on the clinical hard endpoints with KBI [1]. A good apposition of the stent at the polygon of confluence and over the side branch (SB) occurs only when performing a correct POT, that is, the distal marker of the balloon should be at the level of the carina of the bifurcation [1,2]. An incorrect POT, i.e., too far behind (too proximal), leads to incomplete expansion at the SB ostium [9]. If later KBI is performed and then re-POT, this malapposition can be corrected and, if not, an excessive amount of metal can remain next to the SB, with an as yet unknown clinical impact. Moreover, an imperfect too proximal balloon position leads to an unfavorable deformation of the stent’s side cell for eventual re-wiring and dilation. This in turn leads to a too proximal re-wiring, with the formation of a metallic neocarina / a stent ring over the SB origin (with distal re-wiring this ring is pushed towards the SB wall) [9].

These hanging stent struts could affect the laminar flow in the SB, and can even reduce the flow in the SB, especially if its ostium is additionally affected or the struts can remain non-endothelialized or thrombogenic. Until now, however, their potential harm remains unknown. This malapposition is often subtle on intracoronary imaging, especially since IVUS is frequently used in LM, which has a lower resolution than OCT, therefore, for this particular measurement, detecting it through stent enhancement technologies may be a better option. 

**Figure 1:**
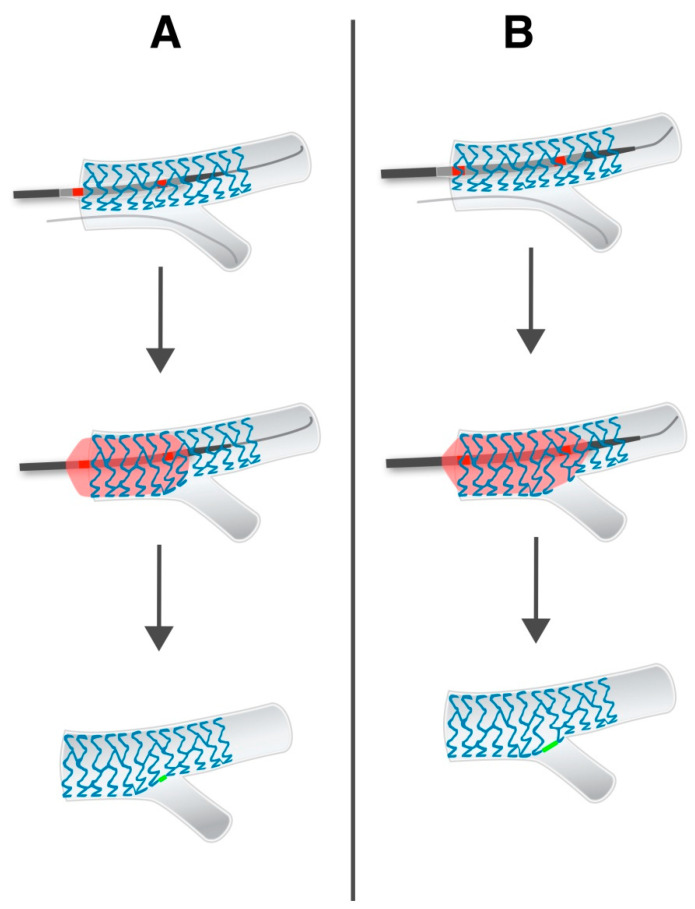
The stent enhancement side branch length (SESBL) concept and how a POT performed too proximal (panel **A**) may cause incomplete stent deformation at this level. (Panel **B**) shows optimal POT positioning with subsequent optimal SESBL. The green line signifies the translucent area seen on stent enhancement.

Frequently using stent enhancement in our center, we have observed a translucent area at the level of SB, after performing POT. We considered the length of this translucent area to be associated with the correct stent apposition at this level. This could be observed without performing KBI (which removes any metal from the SB ostium) and the explanation lies in the fact that POT was indeed performed correctly (Figure 1). To our knowledge, this novel sign has never been described and could be a marker for procedural success. It could have clinical and procedural relevance if its length is associated with cardiovascular events or SB compromise. This is of particular importance especially when choosing not to perform KBI or verify the apposition with intracoronary imaging. The measurement of this length (stent enhancement side branch length = SESBL) is actually an equivalent of a correctly performed POT and has the potential to be a new marker in this regard. That is, a correct POT equals a longer SESBL. The aim of this study was to analyze if suboptimal SESBL (under 2 mm) correlates with major cardiovascular events, low FFR values (<0.8) in the SB and if there is any difference in outcomes between KBI vs. only POT.

## 2. Methods

### 2.1. Study Design and Population

The SESBL study was a retrospective, single-center, observational study of patients with LM lesions who underwent LM bifurcation PCI between January 2019 and December 2020. In total, 201 patients were consecutively enrolled. All patients underwent PCI with the 1-stent strategy without additional SB stenting. Inclusion criteria were patients with angiographic evidence of a significant LM lesion (stenosis of at least 70% diameter at one or both branches) and clinical indication for PCI with stent implantation. POT and stent enhancement was performed in all patients. The patients who underwent the 2-stent strategy were excluded, as the SB stent is a confounding factor for SESBL-related outcomes. Other exclusion criteria were any contraindication for PCI or PCI without stent implantation. Patients were recruited irrespective of performing KBI but they were divided into two subgroups and analyzed separately. This study complied with the Helsinki Declaration and all patients gave signed informed consent.

### 2.2. The PCI Procedure

All patients underwent percutaneous LM coronary artery revascularization. PCI was performed by using only drug-eluting stents (DESs). Baseline and angiographic characteristics of the patients were assessed. Medina classification was used to classify the bifurcation lesions. The procedure was performed by only experienced interventionalists from a high-volume, tertiary center. All decisions regarding the appropriate revascularization strategy were left to the experienced operator. Generally, the LM and left anterior descending (LAD) artery were usually considered as the main branch, and the left circumflex (LCX) artery was regarded as the SB.

The two orthogonal angiographies selected in the post-stenting, post-POT phase were repeated after stent implantation in order to evaluate the SB ostium. Intracoronary imaging was encouraged. Intravascular ultrasound (IVUS) and optical coherence tomography (OCT) were performed at the level of the distal LM bifurcation; their use remained at the discretion of the operator who performed the procedure. Fractional flow reserve (FFR) at the level of the SB was measured in all patients. 

### 2.3. Data Collection, QCA Analysis and SESBL Measurement

A cut-off value of SESBL of 2.0 mm was chosen based on minimal stent area (MSA) cut-off values for the prediction of angiographic in-stent restenosis (ISR) on a segmental basis, which in LCX is >5.0 mm2, meaning a minimum diameter of 2.5 mm at the level of the ostium. As the SESBL is clearly smaller than the actual SB ostium, we arbitrarily chose a smaller threshold. 

**Figure 2:**
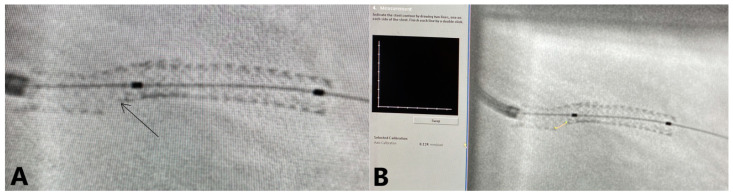
The SESBL measurement process. The arrow (Panel **A**) depicts the translucent area that is to be measured in (Panel **B**).

All cines were reviewed and analyzed blindly by 2 investigators. Due to subjective differences, a pre-specified measuring protocol was defined. SESBL was defined as the translucent length measured at the level of the SB, overlapping the quantitative coronary analysis (QCA) over the stent enhancement images. The software was calibrated with the diameter of the catheter and then the investigator could draw the contour of the SESBL. For precision, the diameter of the stent (which was already known) was also verified (Figure 2).

We used StentBoost (Philips Medical Systems) to enhance the stent visualization angiographic technique. After stent deployment and balloon deflation, an enhanced stent image (ESI) is produced from a minimum of 20 cine frames over 3 s using the radiopaque markers of the delivery balloon as an anchor to align the stent across all frames. The StentOptimizer system automatically grabs the cine images to create a still image of the stent with enhanced edges and the associated region of interest.

All angiographic images were obtained with a digital flat-panel cardiac imaging system (Allura Xper FD 20, Philips Medical Systems). Analysis was performed by validated and automated edge-detection software in all patients in two orthogonal views.

### 2.4. Study Outcomes 

All patients were followed up for one year by reviewing their hospitalization medical records, outpatient return visits and telephone follow-ups. The primary endpoints were the major adverse cardiovascular events (MACEs) that occurred during the patients’ hospitalization and follow-up, including TLR, MI and cardiac death. The secondary endpoints were FFR at SB < 0.80 and difference in MACEs with KBI vs. no KBI. 

### 2.5. Statistical Analysis

Continuous variables were expressed as mean ± standard deviation of the mean, and were compared by use of the unpaired *t*-test; categorical variables were compared with the χ2 statistics or Fisher exact test. The Kolmogorov–Smirnov test was used to test the null hypothesis that a set of data comes from a normal distribution. The cumulative incidence of clinical events is presented as a Kaplan–Meier estimate, and the significance level was assessed with a log-rank test. Hazard ratios (HRs) and 95% confidence intervals (CIs) were calculated using Cox proportional hazard models. Results were depicted by using the corresponding diagrams. Statistical analysis was performed with MedCalc Statistical Software 19.6.1 (MedCalc Software Ltd., Ostend, Belgium; http://www.medcalc.org; accessed on 10 January 2023). A *p* value of <0.05 was considered significant.

## 3. Results

The study included 201 patients who underwent revascularization using a “provisional” technique for LM bifurcation stenosis, using either a one- or two-stent technique. They had a mean age of 68.2 ± 5 years, and 69% were male. All the patients presented several cardiovascular risk factors, among which arterial hypertension was the most frequent one (82%). Clinical presentation was an ST-elevation acute myocardial infarction in 7 patients (3.5%), non-ST-elevation acute coronary syndrome in 13 patients (6.5%) and unstable angina in 85 patients (42%). The rest of the patients (n = 105, 52%) were elective patients. The number of admission days was 3.5 ± 1. Five patients needed inotropic or vasopressor medication during admission, but none needed intraaortic balloon pump therapy. The mean Syntax Score was 18.5 ± 9. The mean number of stents implanted in one patient was 1.1 as 24 additional stents were implanted due to various technical reasons (geographical miss, edge dissection, two contiguous stents from the distal main branch to the LM, etc.). 

Out of these patients, 162 were revascularized with the one-stent LM PCI technique. They had a mean age of 66.8 ± 9.9 years, and 71% were male. All the patients presented several cardiovascular risk factors, among which arterial hypertension was also the most frequent one (91.3%). The clinical presentation was ST-elevation acute myocardial infarction in 5 patients (3.1%), non-ST-elevation acute coronary syndrome in 9 patients (5.6%) and unstable angina in 68 patients (42.2%). The rest of the patients (n = 80, 49.4%) were elective patients. LVEF was 49.4 ± 7.6%. All the bifurcation disease cases involved the left main vessel (100%), and all of these patients received a one-stent PCI technique (n = 162, 100%). The mean SESBL was 2.0 ± 1.2 mm. Many bifurcations had both main vessel (proximal and distal segments) and side branch lesions (Medina 1-1-1) (84 patients, 51.9%). Table 1 summarizes both the general characteristics and the selected treatment strategy for each bifurcation and the QCA results, with emphasis on the SB characteristics. Although half of the cases were true bifurcations (SB disease), the length of disease was short (5.2 ±1.8 mm). This allowed a provisional one-stent technique for the studied cohort. Compared to the mean SB diameter, SESBL covered 64.5% of the area at this level (3.1 mm SB mean diameter vs. 2.0 mm SESBL mean diameter). KBI significantly improved SESBL (1.7 mm mean SESBL in the non-KBI group vs. 2.3 mm mean SESBL in the KBI group, *p* = 0.04).

Results were quantified by using FFR and the minimum stent diameter by StentBoost. Mean values of FFR of the side branch were 0.7 ± 0.47 and 111 patients (68.5%) showed values of FFR > 0.8. One hundred and thirty-five patients (83.3%) had a SESBL > 2 mm, with mean values of 2.0 ± 1.2 mm. The ROC curve analysis showed FFR to predict SESBL values above 2 mm (the “area under curve” of FFR (0.61, 95% CI 0.525–0.690, *p* = 0.036) with the optimal cut-off value of >0.86, which provided Se = 74.5% and SP = 47% for the prediction of SESBL values > 2).

**Figure 3:**
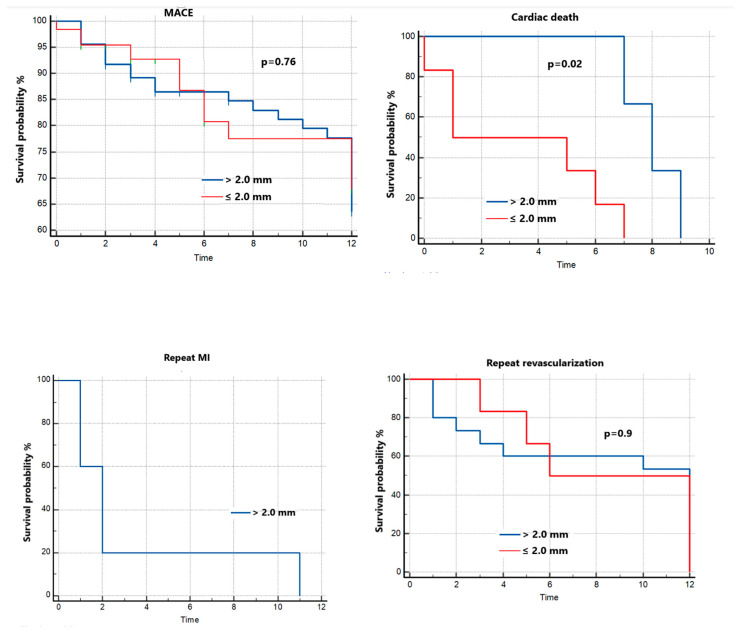
Kaplan–Meier Curves for Clinical Outcomes according to SESBL. MACE = major cardiovascular event, MI = myocardial infarction.

The Youden index for SESBL was determined by ROC curve analysis and the cut-off value was used to determine survival at 1 year. A cut-off value of ≤2.3 was associated with the occurrence of MACEs (AUC = 0.63, Se = 75%, Sp = 56.3%), but the association was not statistically significant (*p* = 0.2). During follow-up (12 months), there was no significant rate of any MACEs in patients with SESBL ≤2.0 mm compared with patients with SESBL >2.0 mm although there was a significantly higher rate of cardiac death in the SESBL ≤2.0 mm group (Figure 3). In terms of stroke and repeat MI, the clinical outcomes were without a significant difference across the two groups.

**Figure 4:**
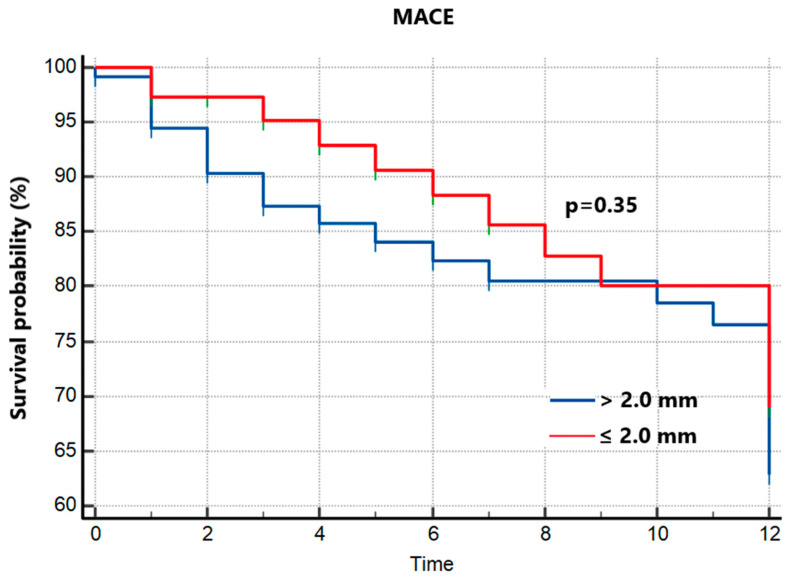
Kaplan–Meier survival analysis for 1-year MACEs according to use of KBI. MACE = major cardiovascular event.

Among the patients who received KBI, the cumulative incidence of MACEa did not significantly differ from the group that received only POT. Figure 4 shows similar clinical outcomes between patients that received KBI and patients that did not (*p* = 0.35). There was also no significant difference in cardiac death (*p* = 0.17), repeat MI (*p* = 0.22), stroke (*p* = 0.30) and repeat revascularization (*p* = 0.15). The significant “visual” difference of SESBL between the non-KBI group and the KBI group did not have an impact on clinical outcomes, only shorter SEBL < 2.0 mm, regardless of performing KBI or not.

## 4. Discussion

The major findings from the present study were the following: (1) the study reconfirmed the major importance of POT during LM PCI, (2) in real-life scenarios where intracoronary imaging is not available or KBI is not feasible, obtaining a SESBL more than 2.0 mm can reassure the operator that the POT was correctly performed, (3) KBI once more does not bring a significant clinical outcome benefit but it may correct a POT performed too proximally (significant difference in SESBL in terms of KBI vs. no KBI), (4) leaving metal at the level of the SB ostium combined with intrinsic ostial disease may hemodynamically compromise the flow at this level. A translucent area at the SB level may show good stent apposition at the level of the polygon of confluence. It must be recognized though that the magnitude of SESBL is directly dependent on the caliber of the SB (as in, a more dominant LCX has a longer SESBL), although after a point, POT cannot increase the length of SESBL, only KBI can enlarge it further. On the other side, a shorter SESBL implies an underexpanded stent at this level. Anatomically speaking, the diameter of the polygon of confluence should be larger than that of the LM body. The previously documented “melon seed” effect of the POT balloon in a funnel-shaped LM may push the balloon out toward the aorta which may lead to a shorter SESBL [10]. This could be avoided with sufficient plaque modification and pre-treatment.

Aggressive post-dilation at the bifurcation may come with the cost of significant carina shift towards the SB and this may further mandate a two-stent bifurcation technique but an underexpanded stent at this level or a compromised SB comes with a mortality hazard. In a recent meta-analysis, Kan et al. found no significant difference between one-stent and two-stent techniques in terms of MACEs but the two-stent approach had a clinical advantage over the provisional strategy when the SB lesion length was >10 mm due to fewer cases of TLR and MI [11]. These findings emphasize the clinical and technical relevance of the SB, which in LM bifurcation is not to be neglected. In consequence, POT is mandatory irrespective of any clinical or anatomical factors while KBI and a second stent play a major role if the SB is large and the plaque incorporates more than its isolated ostium. These affirmations are consistent with our study as in our Medina 1-1-1 patients the length of the SB disease was short and all patients presented good outcomes with the provisional approach. SESBL therefore remains a useful interventional measuring tool only in this subset of patients.

Other aspects that were not investigated in this study are the angle of the LM bifurcation and the morphology of the atherosclerotic plaque (calcific, lipidic, calcified nodule, etc.). Additionally, there are contemporary methods of KBI with a drug-eluting balloon in the SB, but our study did not include such patients and, currently, a head-to-head comparison study does not exist.

**Figure 5:**
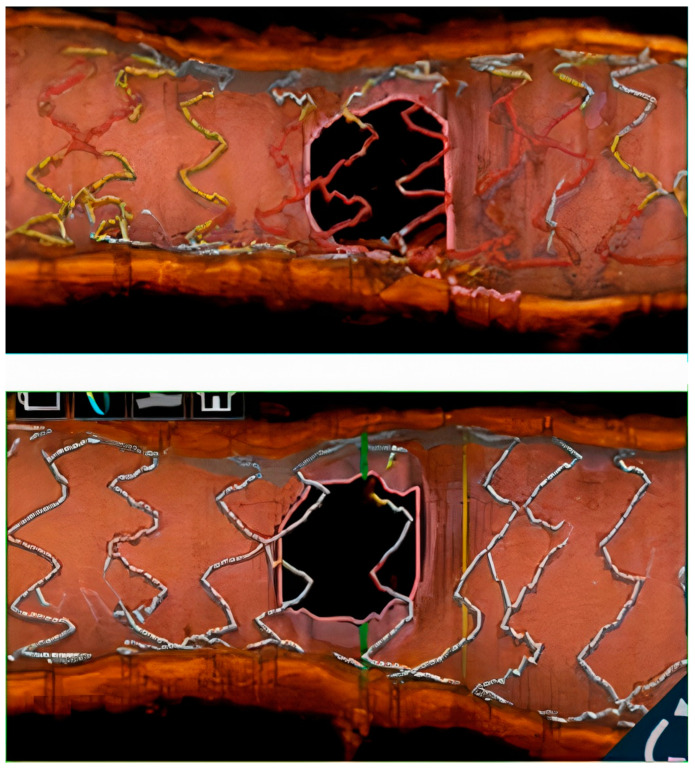
The effect of POT on the stent struts next to the ostium of the SB (before and after POT).

The fact that, in our study, intracoronary imaging was relatively little used is consistent with our findings because, retrospectively, shorter SESBL could have been avoided with more intracoronary usage. Shorter SESBL correlates to underexpansion at the level of the bifurcation, which can be observed and addressed with intracoronary imaging. Verifying before stenting with IVUS or OCT if the lesion has been sufficiently prepared and post-stenting if there is underexpansion at this level could lead to additional optimization steps that implicitly lead to a better SESBL. In addition to these principles, the authors believe that KBI also brings a benefit even if in the current study it did not have an impact on clinical outcomes. In fact, other studies have also shown the lack of a clear benefit on clinical hard-points of KBI, but from a technical and flow dynamics perspective, some benefits have been proven and experts recommend this step in anatomies that allow it [12,13,14]. Indeed, the positive impacts of using KBI are better flow dynamics, it eliminates floating stent struts and it restores the anatomical shape of the bifurcation, but in the end, an optimally performed POT could almost completely eliminate stent struts from the SB ostium [15]. Figure 5 depicts this concept and, in fact, the POT complements KBI, and it does not eliminate its role, because the re-wiring of the SB becomes easier and aiming for a distal strut improves the post-KBI result. All these findings can be controlled by intracoronary imaging, which takes on multiple essential roles.

No similar studies described the concept of SESBL but the notion of SB compromise after provisional stenting is not new. In a computer simulation study, Iannaccone et al. described an ovalization of the SB ostium that might appear as a significant stenosis on two-dimensional angiography, although the SB ostium area was preserved [16]. These were Medina 1-1-1 true bifurcation simulated lesions [16]. In real-life Medina 1-1-1 bifurcations, Hakim et al. found a 30% rate of SB injury (with FFR < 0.75) after performing POT [17]. More than 60% of their cohort included Medina 1-1-1 bifurcations but they excluded patients with SB disease length > 10 mm [17].

The utility of stent enhancement (StentBoost) to guide PCI for bifurcation lesions was intuitively indicated in research reported a decade prior. The authors noted that angiography alone frequently does not provide adequate visualization of stent deformation or incomplete stent expansion at the ostium of the side branch [18]. With the SESBL concept, stent enhancement techniques may be advantageous since they can evaluate the stent’s total deployment and identify underexpansion or improperly treated lesions. Moreover, with current live stent enhancement techniques, this field is on the verge of technological breakthroughs because the SB location can be evaluated even more clearly [19]. Stent enhancement techniques will not be replaced by intracoronary imaging and the two methods will always be complementary. Particularly given that intracoronary imaging cannot offer real-time information while placing gear inside the coronary arteries, the steps of LM PCI can be more carefully controlled. Most essentially, during live stent enhancement imaging, contrast administration may be applied to direct the POT balloon into the optimal location [20,21]. Stent architecture assessment is also important in order to assess PCI results and complications [22]. Potential applications of stent enhancement are: stent underexpansion (it can show suboptimal stent conformation), damage to stent struts (stent deformation), stent overlap (important when implanting a second stent), stent failure (when treating a lesion in a previously stented vessel), aorto-ostial lesions (adequate ostial coverage) and bifurcations (two-stent techniques, adequate POT, proximal vs. distal SB re-wiring or, as in our case, SESBL assessment) [23]. Imaging will aid operators in lesion preparation, stent selection and stent optimization with post-dilation or extra stent implantation [24,25,26]. Longitudinal stent deformation is particularly easy to notice during enhanced stent visualization [24]. Catheterization laboratories should make a determined effort to incorporate imaging into their routine PCI practice and submit data on imaging usage during PCI [27,28] and stent enhancement should always be perceived as complementary to intracoronary imaging and not a surrogate.

There are a number of limitations in the current work that should be considered as caveats. This was a single-center study and, although the sample size was relatively large, the validity of SESBL must be tested by other clinical studies, in a wider integration of stent enhancement roles. All major cardiovascular events were studied up to 12 months. SESBL measurement is dependent on the clarity of stent enhancement acquisitions and the clinician measured this length manually, although we have implemented a standard protocol to minimize these potential limitations. Significant noise interference is increased in heavily calcified vessels and segments with multiple stents and some ultrathin stent platforms are hardly seen on ClearStent/StentBoost, but in our study all ambiguous acquisitions were eliminated. Although correlated with MACEs, the accuracy of SESBL remains to be determined in other studies (MACEs correlated with SESBL may be biased due to other procedural issues such as stent expansion with final minimum stent area, bystander coronary artery disease, patient comorbidities, etc.). Intracoronary imaging and the invasive determination of FFR are more sensitive in assessing the impairment of the SB ostium and they are already established methods.

## 5. Conclusions

Suboptimal SESBL is correlated with worse outcomes and SB compromise. This novel sign could aid the LM operator to assess the level of stent expansion at the ostium of the side branch in the absence of intracoronary imaging.

## Figures and Tables

**Table 1:** General and LM bifurcation lesion characteristics of patients who underwent revascularization using 1-stent technique.

Variable	Value
**General characteristics**	
Age, years (mean ± SD)	66.8 ± 9.9
Male gender (n,%)	115 (71)
Arterial hypertension (n,%)	147 (91.3)
Obesity (n,%)	47 (29.2)
Smoking (n,%)	21 (13.1)
Dyslipidemia (n,%)	137 (85.1)
Diabetes (n,%)	60 (37.3)
LVEF, % (mean ± SD)	49.4 ± 7.6
**Preprocedural characteristics**	
LM bifurcation, n (%)	162(100%)
Provisional 1-stent technique, n (%)	162 (100%)
Only POT, n (%)	147 (90.7%)
Kissing balloon inflation, n (%)	49 (30.2%)
Number of stents implanted, n (%)	162
Number of stents per patient, n (%)	1.1
Intracoronary imaging, n (%)	43 (26.5%)
Medina classification, n (%)	
1-1-1	84 (51.9%)
1-0-1	16 (9.9%)
0-1-1	7 (4.3%)
1-0-0	8 (4.9%)
1-1-0	22 (13.5%)
0-1-0	10 (6.2%)
0-0-1	15 (9.3%)
**QCA**	
Stenosis pre-PCI, % (mean ± SD)	83.3 ± 10.7
MV reference diameter, mm (mean ± SD)	4.4 ± 1.3
SB reference diameter, mm (mean ± SD)	3.1 ± 0.5
SB ostium stenosis > 50%, n (%)	84 (52%)
SB lesion length, mm (mean ± SD)	5.2 ± 1.8
SESBL length, mm (mean ± SD)	2.0 ± 1.2
LM area IVUS/OCT, mm^2^ (median [IQR])	4.2 [3.6–5.2]
FFR side branch (mean ± SD)	0.7 ± 0.47
Number of patients with FFR > 0.8 (n, %)	111 (68.5)
Number of patients with SESBL > 2 mm (n, %)	135 (83.3)

SESBL, stent enhancement side branch length; FFR, fractional flow reserve; IABP, intraaortic balloon pump therapy; IVUS, intravascular ultrasound; LM, left main; LVEF, left ventricular ejection fraction; MV, main vessel; OCT, optical coherence tomography; PCI, percutaneous coronary intervention; POT, proximal optimization technique; SB, side branch.

## Data Availability

The data that support the findings of this study are available from the corresponding author, upon reasonable request.

## Abbreviations

PCI	percutaneous coronary intervention
LM	left main
POT	proximal optimization technique
KBI	kissing balloon inflation
SESBL	stent enhancement side branch length
IVUS	intravascular ultrasound
OCT	optical coherence tomography
LAD	left anterior descending artery
LCX	left circumflex artery
FFR	fractional flow reserve
